# Early biological reactions to a novel hybrid knit fabric inducing autologous tissue ingrowth implanted in a small infant

**DOI:** 10.1186/s44215-026-00275-0

**Published:** 2026-09-08

**Authors:** Takashi Kido, Shintaro Nemoto, Jun Narita, Hidekazu Ishida, Ryo Ishii, Takayoshi Ueno, Shigeru Miyagawa

**Affiliations:** 1 Department of Cardiovascular Surgery, The University of Osaka Graduate School of Medicine, Osaka, Japan; 2 Department of Thoracic and Cardiovascular Surgery, Osaka Medical and Pharmaceutical University, Osaka, Japan; 3 Department of Pediatrics, The University of Osaka Graduate School of Medicine, Osaka, Japan

**Keywords:** Congenital heart surgery, Surgical material, Tissue regeneration, Biodegradable polymer

## Abstract

**Back ground:**

Patch augmentation of the right ventricular outflow tract (RVOT) is frequently required in pediatric patients with stenosis or atresia affecting the right ventricle-to-pulmonary artery continuity. However there have been no ideal patch materials for pediatric patients.

**Case presentation:**

A 1-month-old infant with pulmonary atresia with an intact ventricular septum underwent surgical pulmonary valvotomy and RVOT reconstruction using a novel hybrid wrap-knitted patch designed for autologous tissue ingrowth. Ten months postoperatively, for recurrent severe pulmonary valve stenosis, the patient underwent repeat RVOT reconstruction. Histological examination of the excised hybrid patch revealed that both surfaces were covered with regenerated tissue composed of smooth muscle cells, blood vessels, and collagen fibers. Transmission electron microscopy confirmed that the regenerated tissue contained feature mature fibroblasts and capillaries, demonstrating sufficient development of regenerated tissue.

**Conclusions.:**

Histological findings of the hybrid wrap-knit fabric patch excised in redo surgery 10 months after the initial surgery showed potential macrophage-fibroblast interaction, promoting tissue remodeling in the new fabric.

**Supplementary Information:**

The online version contains supplementary material available at https://doi.org/10.1186/s44215-026-00275-0.

## Background

Patch augmentation of the right ventricular outflow tract (RVOT) is frequently required in pediatric patients with stenosis or atresia affecting the right ventricle-to-pulmonary artery continuity. Current patch materials, including autologous or xenograft pericardium, homografts, bovine jugular vein, expanded polytetrafluoroethylene, and various extracellular matrices, lack growth potential and are prone to calcification over the long term. These limitations complicate the postoperative course in pediatric patients [1].

To address the burdens of existing products, a novel hybrid fabric (SYNFOLIUM^®^, Teijin Medical Technology Inc. Osaka, Japan) was developed as an alternative cardiovascular surgical patch. This material is knitted from a combination of bioabsorbable poly L-lactic acid (PLLA) and nonabsorbable polyethylene terephthalate (PET) yarns, coated with cross-linked gelatin [2]. Following implantation, the bioabsorbable component gradually degrades to facilitate autologous tissue ingrowth. The stretchable structure of the remaining nonabsorbable knit [3] is designed to accommodate physical growth. This process of in situ tissue regeneration has the potential to mitigate the inflammatory and foreign body reactions, and cell death, leading to implant deterioration.

SYNFOLIUM^®^ received regulatory approval in Japan on July 12, 2024, and has been used in both pediatric and adult patients with congenital heart disease [4, 5]. Here, we describe the initial clinical use of this hybrid knit fabric for RVOT enlargement in an infant with pulmonary atresia and intact ventricular septum (PA/IVS). Due to recurrent severe pulmonary valve stenosis 10 months postoperatively, the patch was explanted during a second surgery and analyzed histologically.

## Case presentation

**Fig. 1:**
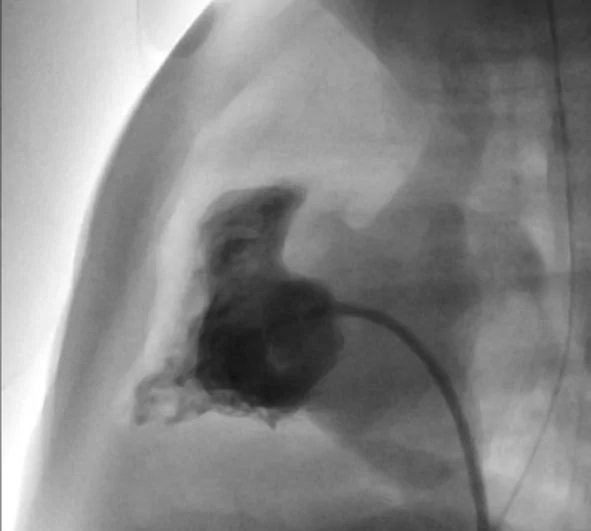
Lateral view from the preoperative right ventricular angiography

**Fig. 2:**
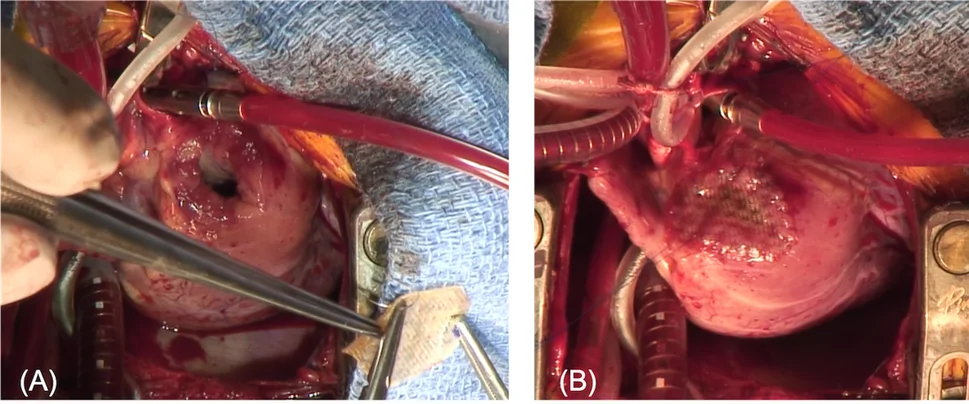
Intraoperative view of right ventricular outflow tract during the first surtegy. **A**. Following pulmonary valve commissurotomy, the right ventricular infundibulum is incised by resecting the excess hypertrophied muscle. **B**. The trimmed hybrid wrap-knit fabric patch is sutured to the incision to enlarge the right ventriculart outflow tract

**Fig. 3:**
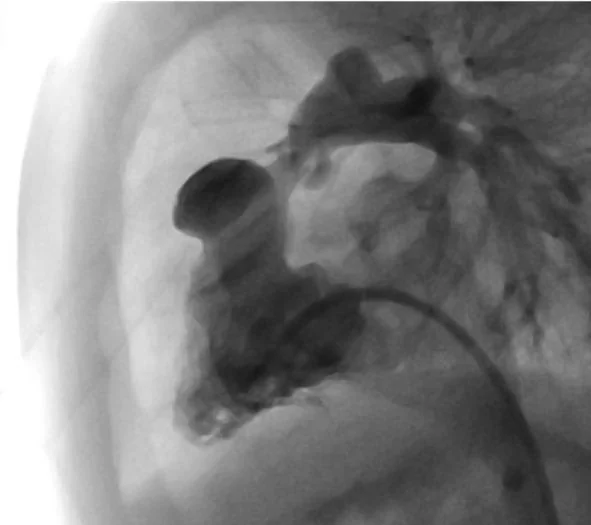
Lateral view of the right ventricular angiography performed 10 months postoperatively

**Fig. 4:**
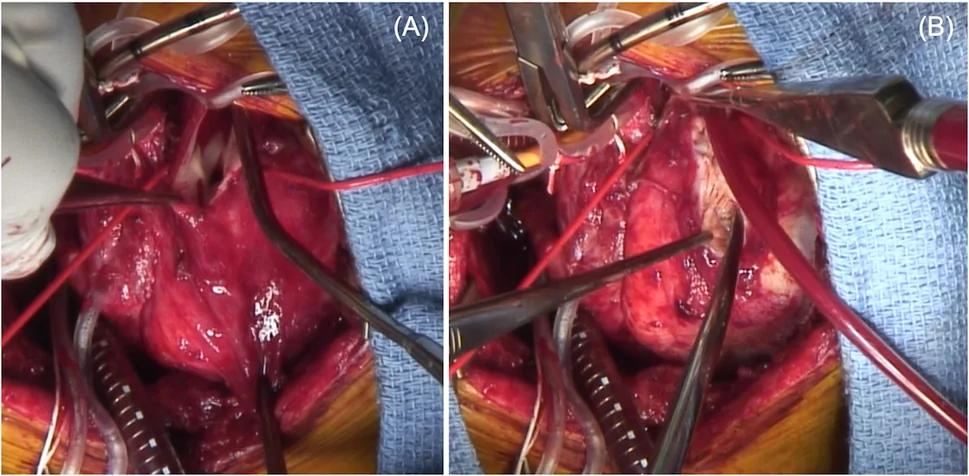
Intraoperative gross view of the hybrid knit fabric patch during the reoperative procedure. **A**. Before removal of the hybrid knit fabric patch. **B**. During the removal of the hybrid knit fabric patch

A 29-year-old female was referred to our hospital at 19 weeks of gestation following a fetal echocardiography diagnosis of PA/IVS. The infant was born at 38 weeks of gestational age with a birth weight of 3.1 kg. A prostaglandin E1 infusion was initiated immediately to maintain ductal patency. Balloon atrial septostomy was performed on the 10th postnatal day. Right ventricular angiography revealed a tripartite right ventricle (RV) with an end-diastolic volume of 4 ml (44% of normal; Fig. 1). Echocardiography showed severe tricuspid regurgitation and a nearly normal-sized tricuspid valve annulus (diameter: 1.19 mm; z-score: 0.61). Computed tomography demonstrated a pulmonary valve annulus diameter of 6.0 mm (z-score: -1.7) and severe narrowing of the RV infundibulum. On the 38th postnatal day, the patient underwent open pulmonary valvotomy, RVOT enlargement, partial closure of the atrial septal defect, and ductus arteriosus ligation. Surgery was performed via median sternotomy under cardiopulmonary bypass with ascending aortic perfusion and bicaval drainage. Following cardioplegic arrest, the right atrium was opened, revealing a thick, dysplastic tricuspid valve with a 9 mm diameter. The pulmonary trunk was opened longitudinally. The atretic pulmonary valve was thin, and the three commissures were fused. The three leaflets of the pulmonary valve were well-balanced. It was predicted that the native pulmonary valve could function adequately if the annulus developed properly after pulmonary valvotomy. A pulmonary valvotomy was performed by making sharp incisions along the fused commissures under direct vision. Following the valvotomy, a 5 mm Hegar dilator (Z-score: -2.3) passed smoothly through the pulmonary valve orifice.　 The RV infundibulum was incised just beneath the pulmonary valve, and the parietal band was partially resected (Fig. 2A). A trimmed hybrid wrap-knitted fabric patch was sutured to the RV incision using continuous monofilament suture (6 − 0 PROLENE, Ethicon Co. Ltd., NJ, USA; Fig. 2B). We left an interatrial communication measuring 4 mm in diameter. Weaning from cardiopulmonary bypass was uneventful. The patient was discharged 41 days postoperatively in stable conditions. However, recurrent severe pulmonary valve stenosis developed 8 months postoperatively, with an echocardiographic peak flow velocity of 5.5 m/sec and a catheterization-derived pressure gradient of 135 mmHg. RV angiography showed the patch-covered outflow tract segment distended immediately proximal to stenotic pulmonary valve annulus and infundibular narrowing (Fig. 3). Consequently, at 10 months of age, the patient underwent repeat RVOT reconstruction, during which the hybrid patch was replaced with a handmade tri-leaflet expanded polytetrafluoroethylene conduit. There was no evidence of thinning, aneurysmal dilation, or structural failure of the explanted hybrid patch. Both surfaces of the patch were covered with autologous tissue, which could be easily detached. (gross findings shown in Fig. 4). The postoperative course was uneventful and the patient was discharged on postoperative day 16.

### Histological findings

**Fig. 5:**
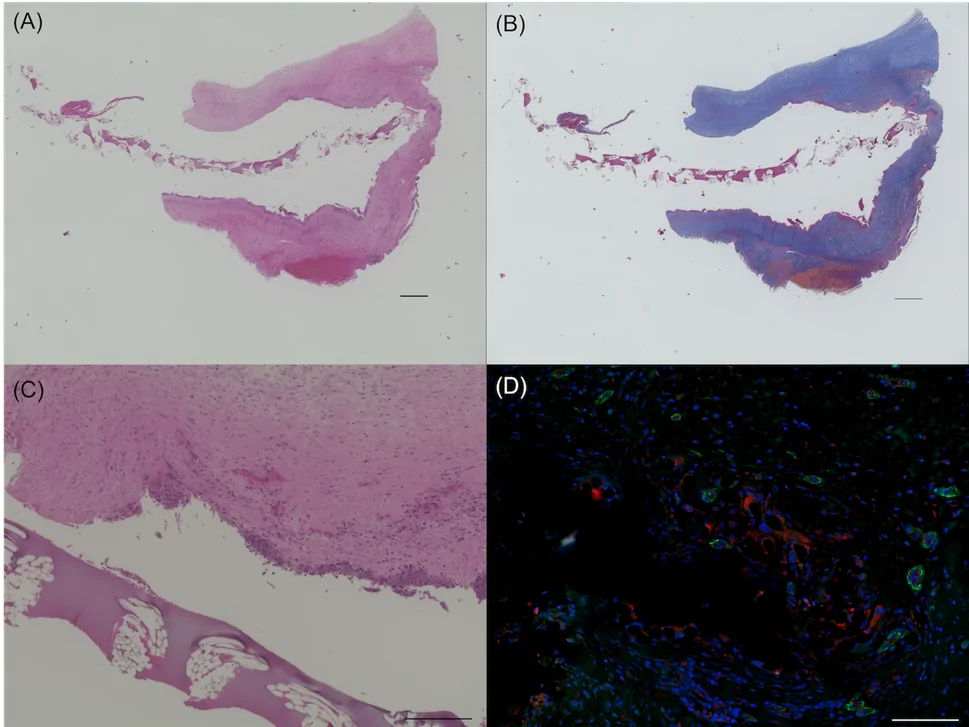
Histological analysis of the explanted hybrid wrap-knit fabric patch and surrounding tissue. Whole-mount views stained with haematoxylin and eosin (**A**) and Masson trichrome (**B**); scale bar = 500 μm, the upper side corresponds to the pericardial surface, and the lower side corresponds to the ventricular surface High-magnification image of the adjunct area between the patch and surrounding tissue are stained with haematoxylin and eosin; scale bar = 200 μm. Representative image of the surrounding tissue is immunostained for alpha-smooth actin (red) and vimentin (green) (**D**); scale bar = 100 μm

Collagen fiber-rich tissue developed surrounding the hybrid patch on its proximal RV side (Fig. 5A and B). Despite a modest infiltration of neutrophils and lymphocytes, no significant chronic inflammation or foreign body reaction to the patch material was observed (Fig. 5C). During the gelatin absorption process, the structural arrangement of all the yarns was preserved (Fig. 5C). Immunohistochemistry revealed smooth muscle cells and small-caliber vasculature within the regenerated tissue (Fig. 5D).

### Transmission electron microscopy findings

**Fig. 6:**
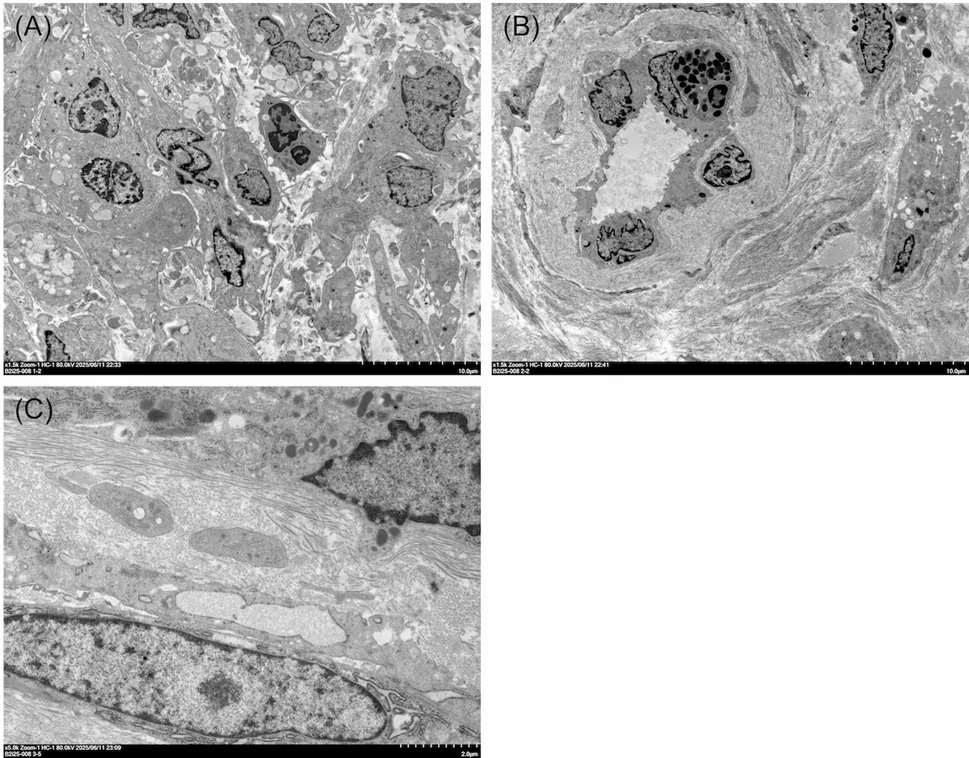
Ultrastructural analysis of the surrounding tissue by transmission electron microscopy. Histiocytic granulomas containing macrophages adjacent to the patch material (**A**); magnification: x 1500. Thick-walled capillaries with endothelial cell and neutrophil infiltration (**B**); magnification: x 1500. Mature fibroblasts exhibiting dilated rough endoplasmic reticulum and increased collagen fibers (**C**); magnification: x 5000

Cardiomyocytes were absent in tissues surrounding the hybrid patch. Histiocytic granulomas containing macrophages were observed adjacent to the patch material (Fig. 6A). The tissue also exhibited thick-walled capillaries with endothelial cells and neutrophil infiltration (Fig. 6B). Mature fibroblasts had a dilated rough endoplasmic reticulum and increased number of collagen fibers (Fig. 6C).

Detailed methodologies for fixation, sectioning, staining, and image capture methods are provided in Supplementary File.

## Discussion and conclusions

Two key findings emerged regarding the early biological response to this novel surgical material in humans. First, the hybrid knit patch functioned without complication under RV pressures exceeding systemic levels. Consistent with RV angiography and intraoperative gross findings, histological analysis revealed no aneurysmal changes, which might have been prevented by the maintained arrangement of all fibers and their attachment to the gelatin. These findings demonstrated material’s safety under systemic pressure, which has not been reported in the previous clinical trial [4]. Second, the autologous tissue formed on patch’s surface was composed of smooth muscle cells, microvessels, and collagen fibers, with only modest inflammatory response, suggesting tissue restoration or regeneration. This contrasts with previous reports on decellularized xenogeneic patches, in which the surface lining is frequently a relatively avascular, densely fibrotic pannus with sustained chronic inflammatory infiltration and calcific degeneration [6], and with expanded polytetrafluoroethylene, which typically elicits a thin, largely acellular fibrous capsule with limited cellular or vascular ingrowth [7]. Electron microscopy confirmed macrophages and mature, collagen-producing fibroblasts, but cardiomyocytes. This cellular profile resembles a local migratory response to myocardial injury that promotes tissue remodeling [8]. In contrast, gelatin absorption was slower than observed in preclinical studies[2, 3]. This delayed absorption may have impeded the formation of bridging tissue, leading to easy detachment of the autologous tissue from the patch. Confirming the long-term efficacy and safety of this hybrid patch for tissue restoration or regeneration will require histological analysis of clinical samples obtained after the complete degradation of the bioabsorbable components.

In conclusion, histological findings of the hybrid wrap-knit fabric patch excised in redo surgery 10 months after the initial surgery demonstrated favorable biocompatibility, structural durability under systemic pressure, and evidence of tissue remodeling through macrophage–fibroblast interaction. Recurrent stenosis in this case appeared to result predominantly from stenotic pulmonary valve annulus rather than from degeneration of the patch itself; these findings support the material’s safety and biological performance but do not establish its efficacy in preventing reintervention, which will require confirmation in larger and longer-term clinical series.

## Supplementary Information

Supplementary Material 1: Detailed methodologies for histological analysis

## Data Availability

All data generated or analyzed during this study are included in this published article.

## Abbreviations

RVOT: Right ventricular outflow tract
PLLA: Poly L-lactic acid
PET: Polyethylene terephthalate
PA/IVS: Pulmonary atresia and intact ventricular septum (
RV: Right ventricle
