# Supplementary material for: Early biological reactions to a novel hybrid knit fabric inducing autologous tissue ingrowth implanted in a small infant
Source: Gen Thorac Cardiovasc Surg Cases. 2026 Sep 8;5:38. doi: 10.1186/s44215-026-00275-0 (PMC13555954; doi:10.1186/s44215-026-00275-0)
Supplement: Supplementary file 1 [file 44215_2026_275_MOESM1_ESM.docx]

Supplementary file.

The excised patch was fixed with 10% buffered formalin for paraffin sections. The paraffin sections were stained with haematoxylin and eosin to examine the general status of the patch and the inflammatory response. In addition, Masson’s trichrome staining was performed to evaluate collagen fibres. Immunohistochemistry with alpha-smooth muscle actin staining and vimentin was performed to detect smooth muscle cells. For transmission electron microscopy, the excised patch was pre-fixed with Karnovsky fixative containing 2.5% glutaraldehyde and 2% paraformaldehyde in a 0.1M(pH 7.4) cacodylate buffer for 2 hours at 4C. The samples were then immersed in 0.5% uranyl acetate for 3 hours at room temperature, dehydrated in ethanol (50%, 70%, 80%, 90%, 95%, and 100%) and propylene oxide, and embedded in epoxy resin. Semithin sections (0.5um) were stained with 0.1% toluidine blue solution and examined under a light microscope. Ultrathin sections were made with a Leica ultramicrotome. These sections were counterstained with uranyl acetate and lead citrate, before examination with a Hitachi H-7100 electron microscope at 75kV.
